# On the Use and Advantages of Opium in the Practice of Obstetricy

**Published:** 1850-08

**Authors:** J. C. W. Lever


*On the Use and Advantages of Opium in the Practice of Obstetricy*. By J. C. W. Lever, M. D.—Perhaps, of all the medicines which our well-endowed Materia Medica contains, there is none more to be valued than opium. Although physiologists may differ as to its mode of action, still both physicians and surgeons will admit that it possesses the power of alleviating anguish, procuring sleep, arresting disease, delaying death. It will be my object to prove that opium should be as highly prized by the midwifery attendant for its therapeutical value in the practice of obstetricy. But so extensive is the subject, that I must, of necessity, for lack of time, and from dread of wearying, direct your attention but to a part of the question. I might treat of its value, in the remedy and cure of some of those signs and symptoms, but which, when severe, are designated diseases, that attend the condition of pregnancy. I might direct your attention to its value in the relief and cure of those maladies, some trivial, many grave, that are wont to develop themselves in the puerperal chamber; but I would rather, on this occasion, speak of the employment and value of opium during parturition; and this division of the subject I have the rather selected because there is much difference in the opinions and in the practice of many gentlemen with whom I have had the pleasure to converse.

And, first, let me investigate ho·v far it is applicable in cases of threatened abortion. Abortion sometimes depends upon the mother, sometimes upon the condition of the ovum itself. The maternal causes of abortion are, for the most part, physical or mental; we must add such as are accidental, those which take place from habit, and those which occur from the effects of poison, as syphilis. But abortion most generally occurs from fetal disease or imperfection; so that the premature emptying the uterus is but an effort of nature to get rid of that which she cannot perfect. In the management of cases of threatened abortion, it is my rule, if possible, to get a thorough knowledge of the immediate or exciting cause of the hemorrhage or pain, or both; secondly, before using opium, to ascertain the state of the os uteri, and especially whether the anterior part of the neck has lost its plumpness and firmness, and has become soft and baggy. If with the discharge we have a patent state of the os uteri, and if the neck be soft and loose, the exhibition of opium will do harm, by retarding the emptying of the uterus, which must sooner or later take place. But while I do not advocate the use of this drug under the circumstances related, I can speak loudly in its praise after the abortion has occurred, especially if such have been attended with a large loss of blood; it will allay excitement, tranquilize the circulation, and procure sleep. These remarks, however, do not altogether apply to those cases which menace from accident, or from mental causes, or those which may be said to be due to habit. In these, with the application of cold, perfect quietude, and unstimulating diet, I have known the exhibition of opium by mouth; or, what I prefer, a cold starch injection, with opium thrown into the bowel, and repeated every night or more often, according to existing circumstances—followed by the best results.

But the value of opium is still more clearly exhibited when it is administered to alleviate those pains which precede the establishment of labor in the latter weeks or months of gestation. Many a patient, by its agency, has been carried on to the full end of term, who but for it would have prematurely parted with her offspring. I had a lady under my care who, six weeks from the completion of her full term, fell on her back. The liquor amnii was evacuated. In addition to absolute quietude, she took opium at irregular intervals till the end of the ninth month, when a living child was born; and this to me was the more satisfactory, as she had on two previous occasions been prematurely confined, although opium had been administered, but with a sparing hand.

Further, we find this drug of great value in certain varieties of natural labor. For instance, at the commencement, there may be irregular and spasmodic pains. They are recognized by their acuteness, by the want of consentaneous action in the uterine fibres; some portion of the uterus during their continuance is hard and contracted, the other portion is soft and yielding; there is no distinct or regular interval of time between the occurrence of pain; and, if untreated or unrelieved, the strength of the patient is exhausted before the establishment of true labor pains; or the child, which at the commencement presented normally with the head, has its position changed to that of the shoulder, by reason of the uterus contracting on one side and forcing its contents over to the uncontracting or yielding side. In such a case, the utility and value of opium are most marked. It may be exhibited by mouth or *per anum.* It will calm the spasm, subdue irregular action, alleviate pain, procure sleep; and after this, true and regular uterine action will be established. Manifold are the instances of its value I have witnessed in such cases.

Not unfrequently in women who marry late in life, and in those who marry very young, do we find the liquor amnii pass away very early—in fact, before the os uteri has commenced to dilate: this may occur spontaneously, or be the result of violence. At all times this is to be regretted; for, in addition to our losing the efficiency of the bag of water to prepare the way for the passage of the child, the fetus is brought into close contact with the uterus, which is therefore more strongly stimulated; the head is brought into direct contact with the os internum, the most sensitive part of the uterus; the labor is more painful, and the birth of a living child is rendered more doubtful. Here the cautious and judicious exhibition of opium controls hyper-uterine action, alleviates pain, and gives a better security for the welfare of the child.

Again, in practice we find women who have suffered in early or unmarried life from one of the forms of dysmenorrhea, when pregnant and in labor, with the os uteri thin, sharp, knife-like, so that its edge is scarcely to be felt—in fact, is often overlooked by the unpracticed finger. The sufferings of the patient are intense; the dilating stage of labor is protracted; and, if untreated or unrelieved, by the time the os uteri is dilated nature is exhausted, uterine effort fails, and such a case is frequently terminated either by the forceps or by craniotomy. In most cases, these evils may be averted by the timely employment of opium, and the best mode of securing its good office is in the form of enema.

Further, we occasionally find the first stage of labor rendered tedious by a hardened, undilatable condition of the os uteri in women who have suffered from chronic inflammation of the neck of the uterus, or those who have worn mechanical contrivances for the purpose of supporting the viscus, and in those who, from disease imaginary or real, have been submitted to the influence of some escharotic, at the present day by far too commonly practiced. This condition of the os uteri needs no description; the sufferings of the patient are excessive and protracted, and, if unrelieved, may be followed by results serious to mother, and fatal to child. In addition to blood-letting, applicable to some cases, to the warm bath, of immense value, to the exhibition of antimony, and this is of the greatest service, we find, when the latter has been exhibited, and has produced its desired results, relaxation of the os uteri, and increase of discharge, that opium given in a full dose will render such permanent, and thus prove a most valuable agent in completing a safe delivery. Opium has been recommended most strongly in cases where the os uteri is callous; but, if the callosity depends upon previous injury, or is the result of disease, its value, in my opinion, depends upon its power to curb uterine action until vaginal interference removes the obstruction to the passage of the fetus. But there is another condition of os uteri in which opium acts, and like a charm: in women who have suffered from irritable uterus where the vagina is generally dry and hot, although not over-sensitive; but the moment the examining finger touches the os uteri, the patient shrieks out, shrinks from the attendant, and by her cries and motions proves the suffering she endures. In addition to subsidiary measures, as the warm bath, the injection of linseed-tea into the vagina, great benefit is to be derived from the use of opium, either by the mouth or by the rectum; the latter mode of employment is the one I prefer. Further, in cases of transverse presentation, where it is necessary manually to interfere to bring the long axis of the child to correspond with the long axis of the uterus, we may assist in relaxing the os uteri, and abate uterine contraction, by the exhibition of a full opiate; but I am no advocate for repeated doses. By such treatment the patient becomes narcotized, uterine efforts arrested, and at the time we need contraction to complete the delivery and prevent hemorrhage, nature fails, and our patient is placed in a situation of extreme peril.

Again, in convulsions, especially those of the hysterical form, occurring as they do more frequently during pregnancy than during labor, opium is a valuable remedy. This form of convulsions, evidencing itself as it does most frequently during gestation, is readily recognized by the predisposition of the patient, often induced by over-fatigue, mental anxiety, irregularity in diet, &c., preceded by intolerance of noise, sleep short and interrupted, twitchings, startings, copious flow of limpid urine, oppression at the chest, difficulty of breathing, globus pain at the upper part or back of the head; and when the convulsions manifest themselves, the larger muscles are more often affected than the smaller; here we find, after the paroxysm is over, that a mild opiate soothes the patient, allays the twitching, calms the respiration, and procures sound and refreshing sleep.

Secondly, in the anemic form of convulsions, associated as they not unfrequently are with large losses of blood, where the face is pale, the eyes glazy, the features shrunken, the countenance betokening exhaustion, the lips colorless, the skin cool, the chest heaving, the breathing labored, the pulse small, quick, and irritable, with noise in the ears, and pain or weight at the top of the head, where is sleeplessness or restlessness, partial amaurosis, strabismus, and sometimes delirium; while close attention is paid to the position of the patient, especially to the position of the head; while stimulants are administed with judgment; while the contraction of the uterus is secured, opium will be found to act like a charm. Again, in genuine eclampsia, where vascular excitement and relaxation of the soft parts have been accomplished by bleeding, purgation, and tartarized antimony, and where the repetition of the fits seems to depend upon irritation, I have seen them occasionally checked by the administration of a full opiate. Labor also may be complicated with tumour; here opium will allay inordinate action until we employ those manual or surgical means which are necessary to remove the obstructing cause to delivery. It is true, opium cannot take away the mechanical obstacle, but it may and will lessen inordinate uterine action; for in practice we find that, if there be any difficulty in the passage of the child, the uterus is stimulated to undue action, and if such be not allayed, or be overlooked, rupture of the viscus itself may take place. This leads me to speak of the efficacy of opium in the treatment of those grave cases where the uterus or vagina is lacerated, or the bladder or diaphragm have ruptured. The two latter lesions are, indeed, to be regarded as all but hopeless; but not the former, for I have seen cases treated by the administration of full doses of opium, and then repeated at varying intervals for several days, and then terminating successfully. To one woman I was called when there was a band in the vagina, the result of a previous delivery; in this case, the laceration was so extensive that the hand could be passed into the abdomen. Although the patient appeared to be dying, although the last rites of her church were administered to her, she rallied, recovered, and is still alive. Soon after her convalescence, she had the ill fortune to lose her husband, but she also had the good fortune not again to be pregnant. But the value of opium is perhaps most emphatically demonstrated when exhibited after floodings, whether such occur in the earlier months of gestation or in the latter, depending either upon the position of the placenta or its partial separation; whether the loss take place after the birth of the child and the casting or throwing off the placenta; whether this be retained by irregular contraction or morbid adhesion; or whether the hemorrhage take place after the complete evacuation of the uterus. In these cases, where there is great exhaustion, alarming syncope, great irritability, severe vomiting, and plain, evident, and undeniable indications of great depression of the sanguiferous and nervous systems, or, to use the graphic language of Dr. W. Griffin: "When the countenance is sunk, the eye hollow and glassy, the lips blanched, the skin cold, and the whole person corpse-like; when the pulse is all but gone at the wrist; when the beat of the heart is scarcely perceptible, and stimulants, even brandy, are vomited or useless, opium will act like magic, and save the patient from an untimely grave; but, to do good, it must be exhibited in full doses of one to two drachms of the tincture, or three to four grains, repeating two grains every half hour or hour until the pulse becomes distinct, the breathing calm, and the jactitation allayed." Whatever may be the "ratio medendi," whether the congestion produced in the brain be what is necessary to maintain the proper tension of the cerebral vessels, whether it restore the loss of nervous power in the brain itself, is still a point "sub judice;" but no man of much obstetric experience will deny its value under the circumstances thus detailed. I could illustrate its efficiency by the recital of several cases of success where the patient appeared to be on the very confines of eternity; and in the subsequent constitutional treatment its exhibition must not be forgotten: it will lessen exhaustion, diminish restlessness, allay vomiting, calm gloomy forebodings, and procure sleep. I say nothing of its employment in instrumental delivery, for so various are the causes that may render inteference necessary, that to go through the whole subject, and select the cases fitted for its agency, would be in this society not only wearisome but unnecessary.—*Lond. Med. Gaz.*, Nov., 1849.

